# War-related displacement, conflict intensity, and treatment delivery factors in opioid agonist maintenance treatment discontinuation following Russia's invasion of Ukraine: a national cohort study

**DOI:** 10.1016/j.lanepe.2026.101831

**Published:** 2026-08-26

**Authors:** Roman Ivasiy, Lynn M. Madden, Anna Meteliuk, Daniel J. Bromberg, Eteri Machavariani, Bachar Ahmad, Tetiana Fomenko, Myroslava Filippovych, Iryna Kharandiuk, Scott O. Farnum, Zahedul Islam, Frederick L. Altice

**Affiliations:** a Section of Infectious Diseases, Yale School of Medicine, New Haven, CT, United States; b Center for Interdisciplinary Research on AIDS, Yale University, New Haven, CT, United States; c APT Foundation, New Haven, CT, United States; d International Charitable Foundation “Alliance for Public Health”, Kyiv, Ukraine; e Ukrainian Institute on Public Health Policy, Kyiv, Ukraine

**Keywords:** Methadone, Treatment retention, Mortality, War, Displacement, Ukraine, Implementation science, Take-home dosing, NIATx

## Abstract

**Background:**

Ukraine has Europe's second-largest HIV epidemic, driven mainly by injection opioid use. Opioid agonist maintenance treatment (OAMT) is the most effective treatment for opioid use disorder (OUD) and a key HIV prevention strategy. Following Russia's invasion of Ukraine on 24 February 2022, continuity of OAMT was threatened. We examined the effects of displacement, war exposure, and treatment delivery factors on OAMT discontinuation during the first two years of the expanded war.

**Methods:**

In this retrospective analysis of a prospective national cohort study, we used individual-level data from Ukraine's national OAMT registry. Adults (≥18 years) with diagnosed OUD receiving OAMT in 252 government-operated clinics as of 23 February 2022 were followed through 1 January 2024. The primary outcome was treatment discontinuation. War exposure was classified using k-means clustering of six standardised regional indicators: air raids, explosions, artillery attacks, internal displacement, occupation days, and frontline days. Regions were categorised as low-intensity, high-intensity, or frontline. Displacement was modelled as a time-dependent variable in frailty-adjusted extended Cox proportional hazards models.

**Findings:**

In total, 17,211 participants were included (14,554/17,211 (84.6%) men; mean age 38.9 years, with standard deviation = 7.2). During follow-up, 5040/17,211 (29.3%) discontinued OAMT and 921/17,211 (5.4%) died. Compared with local patients in low-intensity regions, discontinuation risk was higher in high-intensity (aHR 1.74; 95% CI 1.40–2.15) and frontline regions (aHR 3.09; 95% CI 2.80–3.42). Internally displaced persons faced markedly elevated risk in low-intensity (aHR 3.55; 95% CI 2.92–4.32), high-intensity (aHR 5.90; 95% CI 4.52–7.69), and frontline regions (aHR 8.26; 95% CI 5.43–12.56). Optimal dosing (aHR 0.74; 95% CI 0.69–0.78) and unsupervised dosing (aHR 0.71; 95% CI 0.64–0.78) were protective. Females had higher discontinuation risk (aHR 1.20; 95% CI 1.11–1.30) than males.

**Interpretation:**

Displacement and residence in high-intensity war regions were dominant drivers of OAMT discontinuation. Flexible dosing policies and continuity strategies for displaced patients are essential to sustain OAMT during war.

**Funding:**

National Institute on Drug Abuse (R01DA033679, R01DA043125, R01DA045384, F31DA054861, R01DA054703).

**Research in context**

**Evidence before this study**

We searched PubMed, Scopus, and Google Scholar from January 1, 2015, to October 1, 2025, using English-language terms including “opioid agonist therapy,” “methadone,” “buprenorphine,” “Ukraine,” “conflict,” “war,” “humanitarian,” “retention,” “natural disaster” and “displacement.” Studies from Eastern Europe and Central Asia (EECA) consistently demonstrate the effectiveness of opioid agonist maintenance treatment (OAMT) with methadone or buprenorphine in reducing HIV transmission and improving antiretroviral therapy adherence and health among people who inject drugs (PWID). Research on OAMT continuity in humanitarian crises, conflict or wartime conditions, however, is scarce. Evidence from other conflict-affected and emergency settings, including Afghanistan, Syria, Myanmar, and regions affected by natural disasters (i.e., Australia, Southern and North-Western U.S. states), demonstrates that OAMT delivery is often disrupted by devastation, restrictive drug policies, and population displacement, leading to treatment interruption, relapse to drug use, and increased HIV transmission and suicide. Prior studies from Ukraine examined OAMT expansion and unsupervised (i.e., take-home) dosing during disruptions from the COVID-19 pandemic and early phases of conflict in 2014 and 2022, but none analysed national data on OAMT retention and discontinuation under conditions of full-scale war, where there was large population displacement, and health system disruption. No prior study incorporated objective conflict exposure metrics or dynamic displacement data into longitudinal survival models of OAMT treatment continuity.

**Added value of this study**

This is the first national study to quantify the impact of war-related displacement and conflict intensity on OAMT retention in a country undergoing full-scale armed conflict. Using data from Ukraine's national OAMT registry, encompassing all government-operated sites, we applied a longitudinal frailty-adjusted Cox model with patient-level random effects to account for repeated treatment episodes. The analysis integrated a data-driven conflict typology, based on k-means clustering of air raids, explosions, artillery attacks, and internal displacement, and linking regional conflict exposure with individual treatment outcomes. Findings show that both displacement and receiving OAMT in frontline regions were the dominant predictors of OAMT discontinuation, while optimal dosing and take-home dosing substantially reduced discontinuation risk. The study also identifies sex- and HIV-related disparities in retention, providing the first empirical evidence of OAMT system resilience and vulnerability during wartime at a national scale.

**Implications of all the available evidence**

Armed conflict and displacement pose major threats to addiction treatment and HIV prevention systems, but optimised OAMT delivery models that rapidly prioritise unsupervised dosing and strategic OAMT dosing can mitigate these risks. The Ukrainian experience underscores that policies enabling unsupervised dosing, cross-regional medication transfer, and adaptive dosing strategies are essential for maintaining continuity of care under crisis conditions. These findings support integrating OAMT into emergency preparedness and humanitarian health frameworks, with emphasis on mobile, low-threshold, and cross-country continuity mechanisms for displaced persons. More broadly, this study provides quantitative evidence that scaling OAMT access and flexibility during humanitarian emergencies is critical to sustaining HIV prevention, reducing overdose risk, suicide and preserving health system resilience in conflict-affected settings.

## Introduction

Ukraine bears the second-largest HIV epidemic in Europe, with nearly one in five people who inject drugs (PWID) having HIV.^1^ Among the estimated 360,000 PWID, 82% have opioid use disorder (OUD), underscoring the importance of opioid agonist maintenance treatment (OAMT) with methadone or buprenorphine for both HIV prevention and treatment engagement.^2^ Across Eastern Europe and Central Asia (EECA), OAMT is among the most effective and cost-effective interventions to reduce HIV transmission among PWID.^3^ Compared with ongoing non-prescribed opioid injection, OAMT reduces injection-related behaviours, reduces HIV transmission, stabilises daily functioning, and improves HIV treatment engagement, while discontinuation of OAMT is associated with transmission risk, overdose mortality and poor HIV treatment outcomes.^4^^,^^5^ Identifying factors associated with treatment discontinuation is therefore critical, particularly during periods of armed conflict and healthcare disruption.

Russia's full-scale invasion of Ukraine in February 2022 created one of the most challenging environments globally for sustaining OAMT.^6^^,^^7^ War, population displacement, and the destruction or occupation of health infrastructure severely disrupted care continuity. In territories newly occupied by invading Russian forces, OAMT and syringe services programs were shut down,^8^ leaving many patients at risk of withdrawal, relapse to drug injection, overdose, suicide, and increased vulnerability to HIV transmission.^9^ In regions that remained under Ukrainian government control, rapid policy and service-delivery adaptations, building on innovations introduced during the COVID-19 pandemic, helped sustain treatment continuity.^7^^,^^10^ Ukraine had already expanded OAMT during the pandemic through increased dosing flexibility, expanded unsupervised (i.e., take-home) dosing (UD), and streamlined treatment delivery, supported in part through implementation strategies including the Network for the Improvement of Addiction Treatment (NIATx).^11^^,^^12^ Following the invasion, these emergency measures were rapidly expanded, including allowing up to 30 days of take-home medication and facilitating cross-regional patient transfer.^6^^,^^11^ During the first year of the war, the number of patients in government clinics increased by 17%, supported by emergency deliveries coordinated with non-governmental organisations.^9^^,^^13^ At least 15.3% of patients were estimated to receive OAMT through private facilities, although the overall scope of private service provision remains uncertain because of limited data availability.^13^

While these adaptations helped sustain OAMT access, the wartime environment increases HIV vulnerability through healthcare disruption, displacement, instability in harm reduction services, and interruptions in care continuity.^9^ Maintaining OAMT during war is especially critical, as it is among the few evidence-based interventions feasible in humanitarian contexts, with mobile and low-threshold delivery models proving effective and acceptable despite resource constraints.^14^ OAMT access, however, remains fragile in many crisis-affected states, undermined by restrictive drug policies, donor transitions, and lack of continuity across borders.^15^ Refugee populations are particularly vulnerable, as interruptions in OAMT have been linked to adverse health outcomes, including overdose mortality and HIV treatment discontinuation.^16^

Despite this knowledge, few studies have provided empirical data from national OAMT programs on how war, including its geographic specificity, impacts retention in HIV prevention interventions. Ukraine offers a unique opportunity to address this gap. Using longitudinal data from Ukraine's national OAMT registry, we aimed to investigate whether displacement, war intensity, and program delivery adaptations were associated with OAMT discontinuation in the first two years of Russia's expanded invasion of Ukraine.

## Methods

### Study design, setting and data sources

We conducted a prospective cohort study using routinely collected national registry data to evaluate continuity of OAMT during the full-scale war in Ukraine. OAMT with oral methadone or sublingual buprenorphine is delivered through government-operated clinics using national treatment protocols.^9^ The study period spanned from February 24, 2022, to January 1, 2024, encompassing nearly two years of Russia's full-scale invasion.

Individual-level data were extracted from *SyRex*, Ukraine's national OAMT registry for publicly-funded OAMT programs.^17^ We included adults aged 18 years or older with a documented diagnosis of OUD who were receiving OAMT on February 23, 2022. SyRex prospectively records enrolment, treatment episodes, inter-site transfers, quarterly updates of dosing history, and unsupervised dosing status for all patients receiving OAMT within government-operated sites. Core demographic, clinical, and treatment-related variables are mandatory reporting fields and records cannot be finalised without completion of these variables, resulting in no missing data for analyses. Registry data are routinely validated by the Ukrainian Public Health Center and Alliance for Public Health.^13^

### Measures

The primary outcome was treatment discontinuation, defined as missing 10 or more consecutive days of OAMT, consistent with national reporting standards. Re-enrolment was defined as return to OAMT following an interruption of at least 10 consecutive days. Patients were censored at death. Medication type was recorded as buprenorphine (BUP) or methadone (MET). OAMT dosage was dichotomised as optimal vs suboptimal using internationally recognised thresholds (≥90 mg/day for methadone or ≥16 mg/day for buprenorphine).^18^ Dosing strategy was classified as unsupervised vs daily supervised dosing. Because dose and dosing strategy were updated quarterly in SyRex, participants receiving treatment before January 1, 2022, were assigned the dose and dispensing status recorded on January 1, 2022. Participants initiating treatment between January 1, 2022, and February 23, 2022 (N = 652, 3.8%), were assigned values from the April 2022 quarterly update. Dose and unsupervised dosing status were modelled as time-varying covariates using the quarterly snapshots (Supplemental Figure S1). By contrast, treatment discontinuation events and inter-regional transfers were recorded using exact dates. Episode type was defined as *initial* (new initiation) vs *subsequent* (reinitiating treatment after a prior discontinuation) OAMT episode. Duration on OAMT at baseline was categorised as ≤30 days, 31–180 days, and >180 days and reflected the duration of the ongoing uninterrupted treatment episode rather than cumulative lifetime exposure across multiple episodes. Sociodemographic and clinical variables included sex, age stratified at the lower quartile (34 years), and HIV status. Duration of drug injection was dichotomised by the lower quartile (13 years).

Additional covariates included regional population size, gross regional domestic product (GRDP) per capita, and baseline regional OAMT program performance. Population size and GRDP were categorised into quartiles based on pre-war baseline regional metrics, while the corresponding covariates were treated as time-dependent to reflect patient mobility across regions during follow-up. Baseline program performance reflected pre-war regional differences in patient acquisition and treatment dropout patterns, derived from a prior national implementation study in which regions were classified into three mutually exclusive categories: high acquisition/low dropout, mid-level acquisition/high dropout, and low acquisition/mid-level dropout (See also Supplemental Material for more details).^19^

### War intensity classification

To capture regional variation in exposure to the war, we constructed a composite oblast-level (region) measure of war intensity using six indicators: 1) cumulative air raid alerts; 2) explosions; 3) artillery attacks; 4) proportion of internally displaced persons (IDP); 5) days of partial occupation; and 6) days of active frontline combat (See Supplemental Table A1). Air raid alerts, explosions and artillery attacks were obtained from the Air Alarms platform, which aggregates real-time official regional emergency alarm data.^20^ Quarterly displacement reports published by the International Organization for Migration were used to obtain IDP estimates.^21^

**Fig. 1:**
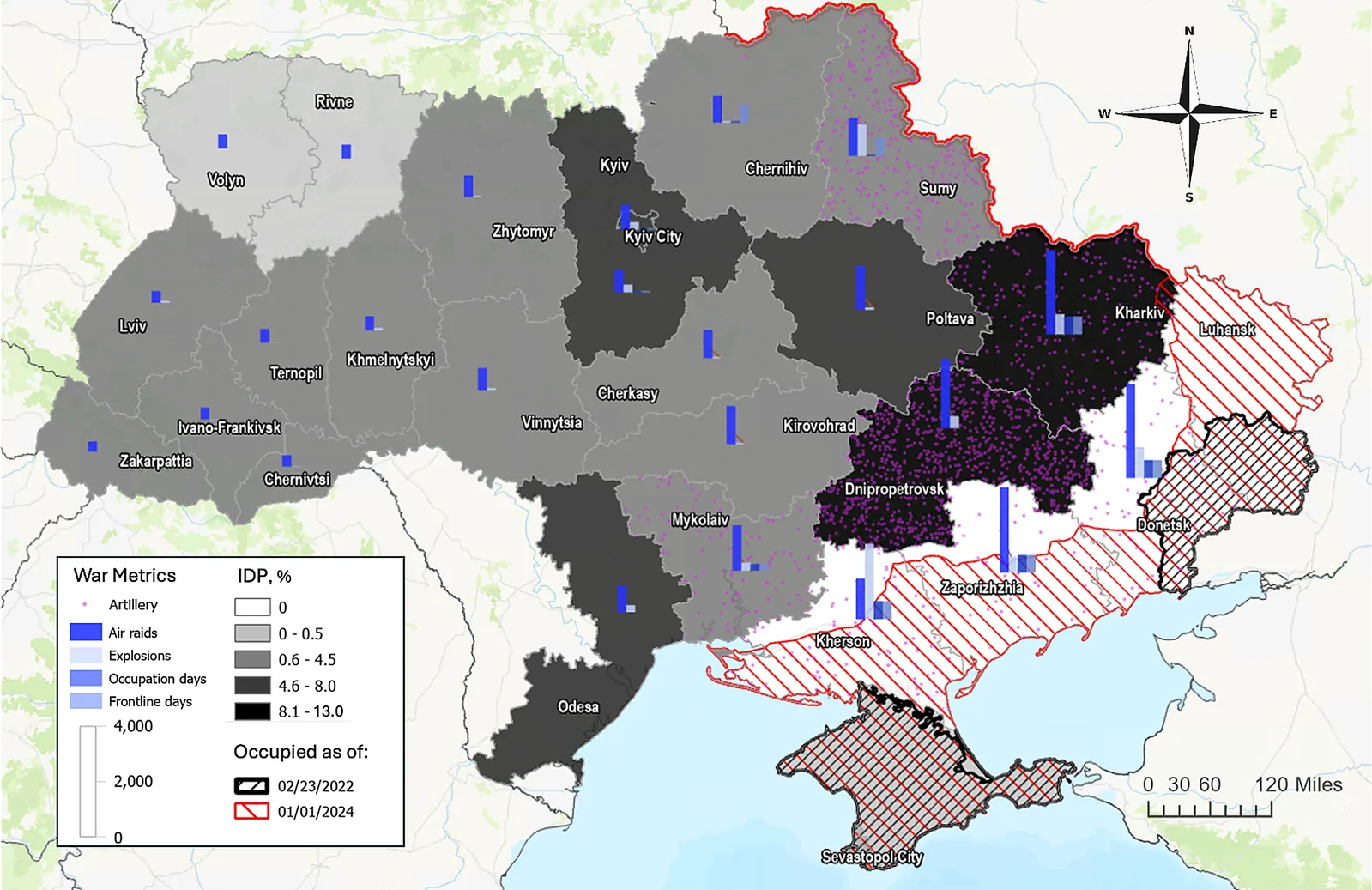
Regional distribution of war exposure metrics and internally displaced persons (IDP) in Ukraine as of January 1st, 2024.

**Fig. 2:**
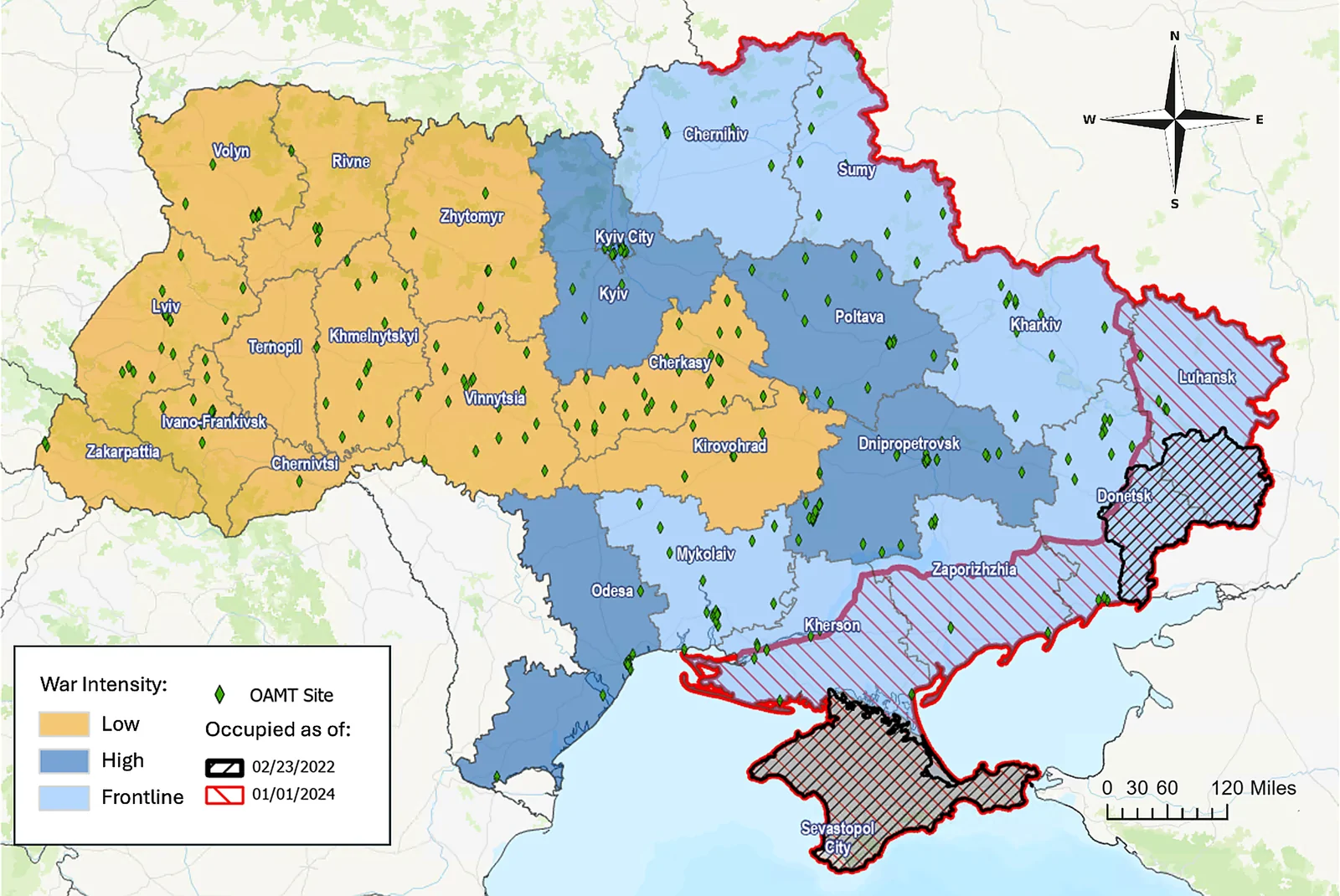
Regional clusters of war exposure in Ukraine derived from K-means analysis of war metrics and internal displacement. **Legend:** OAMT: opioid agonist maintenance treatment.

We applied k-means clustering to standardised war exposure indicators and identified natural groupings of oblasts according to war intensity (Fig. 1; Supplemental Material: Table A1). The optimal cluster structure was evaluated using the gap statistic method and additional validation metrics, which supported a three-cluster solution with acceptable separation and stability (Supplemental Material: Figure A2). The resulting categories included: 1) low-intensity regions characterised by limited direct hostilities and no occupation; 2) high-intensity regions characterised primarily by frequent air raids, missile attacks, minimal occupation and elevated internal displacement; and 3) frontline regions characterised by prolonged active combat (proximity to the frontline), occupation, and extensive artillery attacks (Supplemental Material: Table A1). The final regional classification is shown in Fig. 2.

Displacement status was proxied by inter-oblast patient transfers. Because inter-regional mobility among OAMT patients in Ukraine was historically rare before the invasion (<0.1% before 2020; Supplemental Material, part III), observed movement during the follow-up was interpreted predominantly as war-related displacement rather than routine migration. Displacement status was modelled as a time-dependent variable reflecting movement into a given oblast and was updated dynamically over follow-up to account for secondary movement and return migration, such that person-time and discontinuation events were attributed according to the participant's location at the time each event occurred. Additional relocation analyses are provided in Supplemental Table S1.

### Statistical analysis

Descriptive statistics summarised baseline patient-level covariates across war-affected regions. Categorical variables were compared using χ^2^ tests. Unless otherwise specified, reported χ^2^ p-values reflect comparisons across regional war exposure groups. To account for multiple treatment episodes within the same patient, we applied a longitudinal frailty survival model with individual-level random effects. Frailty models extend the Cox proportional hazards framework by introducing a random effect term (“frailty”) that captures unobserved heterogeneity across patients and appropriately adjusts for the correlation of repeated episodes within the same individual. This approach allows for more accurate estimation of hazard ratios when patients contribute more than one treatment episode. The proportional hazards assumption was evaluated using Schoenfeld residual tests and visual inspection of residual plots, with full diagnostic results provided in the Supplemental Material. Stratified analyses additionally evaluated participants according to their region of residence at the onset of the war (low-intensity, high-intensity, and frontline regions). Multivariable frailty survival models estimated hazard ratios (HRs) with 95% confidence intervals (CIs). The primary model was repeated using a 30-day definition of OAMT dropout as a sensitivity analysis (Supplemental Table S2). Analyses were conducted using SAS version 9.4 (SAS Institute, Cary, NC, USA) and R version 4.3.2 (R Foundation for Statistical Computing, Vienna, Austria), including the *survival* and *frailtypack* packages. Maps were created using ArcGIS Pro 3.3 (Esri, Redlands, CA, USA).

### Ethical approval

The study was approved by the Institutional Review Board of Yale University (IRB Protocol No. 2000021361) and the Institutional Review Board of the Ukrainian Institute on Public Health Policy (Approval No. 00007612). The requirement for informed consent was waived because the study used de-identified routinely collected registry data.

### Role of the funding source

The funder had no role in study design, data collection, data analysis, data interpretation, or writing of the report. The corresponding and the senior authors had final responsibility for the decision to submit the manuscript for publication.

## Results

As of February 23, 2022, 17,211 patients receiving OAMT in government-operated clinics were categorised as residing in low-intensity (4631, 26.9%), high-intensity (6998, 40.7%), and frontline regions (5582, 32.4%) (Table 1). Most participants were men (84.6%), aged ≥34 years (70.7%), and 35.1% were people with HIV. HIV prevalence was highest in high-intensity regions compared with frontline and low-intensity regions (39.8% vs 31.8% vs 32.1%, respectively; p < 0.001). During follow-up, 5040 (29.3%) of participants experienced at least one treatment discontinuation event, 1171 (6.8%) had multiple treatment episodes, and 921 (5.4%) died. From February 24, 2022, through January 1, 2024, 519 patients (3.0%) relocated internally at least once. Two marked increases in discontinuation occurred in March 2022 and July 2022 within frontline regions, temporally corresponding to intensified Russian offensives in Donbas in the East and Kherson in the South of Ukraine, and periods of regional occupation (Supplemental Table S1).

**Table 1:** Baseline sociodemographic and clinical characteristics of opioid agonist maintenance treatment patients by regional conflict exposure cluster in Ukraine (N = 17,211).

Characteristic	Region cluster						Total		Chi-square p-value
	Low-intensity		High-intensity		Frontline				
	N	%	N	%	N	%	N	%	
**Total**	4631	26.9	6998	40.7	5582	32.4	17,211	100	
Age (stratified by lowest quartile), years									
<34	1605	34.7	1862	26.6	1582	28.3	5049	29.3	<0.001
≥34	3026	65.3	5136	73.4	4000	71.7	12,162	70.7	
Sex									
Male	4090	88.3	5726	81.8	4738	84.9	14,554	84.6	<0.001
Female	541	11.7	1272	18.2	844	15.1	2657	15.4	
Dosage									
Optimal	2284	49.3	3196	45.7	3116	55.8	8596	49.9	<0.001
Suboptimal	2347	50.7	3802	54.3	2466	44.2	8615	50.1	
Medication									
Methadone	3904	84.3	6284	89.8	4971	89.1	15,159	88.1	<0.001
Buprenorphine	727	15.7	714	10.2	611	10.9	2052	11.9	
Dosing									
Daily supervised	705	15.2	988	14.1	937	16.8	2630	15.3	<0.001
Unsupervised	3926	84.8	6010	85.9	4645	83.2	14,581	84.7	
Episode type									
First episode	3686	79.6	5746	82.1	4631	83	14,063	81.7	0.002
Not first episode	945	20.4	1252	17.9	951	17	3148	18.3	
OAMT duration									
≤30 days	100	2.2	188	2.7	132	2.4	420	2.4	<0.001
31–180 days	303	6.5	654	9.3	469	8.4	1426	8.3	
>180 days	4228	91.3	6156	88	4981	89.2	15,365	89.3	
HIV status									
HIV-negative	3144	67.9	4213	60.2	3806	68.2	11,163	64.9	<0.001
HIV-positive	1487	32.1	2785	39.8	1776	31.8	6048	35.1	
Drug injection duration (stratified by lowest quartile), years									
<13	1581	34.1	1672	23.9	1504	26.9	4757	27.6	<0.001
≥13	3050	65.9	5326	76.1	4078	73.1	12,454	72.4	
Pre-War population quartile, baseline									
0.9–1.0 million	908	19.6	0	0	1779	31.9	2687	15.7	<0.001
1.1–1.2 million	1994	43.1	0	0	1146	20.5	3140	18.3	
1.4–2.1 million	972	21	1620	23.1	1216	21.8	3808	22.1	
2.4–4.1 million	757	16.3	5378	76.9	1441	25.8	7576	44	
Pre-War GRDP per capita quartile									
€0.5 k–1.7 k	634	13.7	0	0	1891	33.9	2525	14.8	<0.001
€1.7 k–2.1 k	1945	42	0	0	719	12.9	2664	15.5	
€2.2 k–2.7 k	2052	44.3	796	11.4	2972	53.2	5820	33.7	
€3.2 k–8.4 k	0	0	6202	88.6	0	0	6202	36	
Pre-war region OAMT performance category									
Low	1197	25.8	2274	32.5	1953	35	5424	31.5	<0.001
Moderate	2168	46.8	3262	46.6	3264	58.5	8694	50.5	
High	1266	27.3	1462	20.9	365	6.5	3093	18	

At baseline, 8596 participants (49.9%) received an optimal OAMT dose, with the highest proportion observed in frontline regions (55.8%; p < 0.001). Methadone was the predominant medication overall (88.1%), whereas buprenorphine use was more common in low-intensity regions (15.7%) than in frontline (10.9%) or high-intensity regions (10.2%; p < 0.001). Unsupervised dosing was widespread, with 14,581 participants (84.7%) receiving take-home medication at baseline. Most participants (81.7%) were in their first OAMT episode, and 89.3% had remained on treatment for at least 180 days before the invasion.

Table 2 presents factors associated with OAMT discontinuation during the first two years of Russia's invasion of Ukraine. Displacement status along with the regional war intensity was the strongest predictor of discontinuation. Compared with local participants in low-intensity regions, discontinuation risk was higher among those residing locally in high-intensity regions (aHR 1.74, 95% CI 1.40–2.15; p < 0.01) and frontline regions (aHR 3.09, 95% CI 2.80–3.42; p < 0.01). Displacement was associated with even greater risk, increasing progressively from participants displaced into low-intensity regions (aHR 3.55, 95% CI 2.92–4.32) to those displaced into high-intensity (aHR 5.90, 95% CI 4.52–7.69) and frontline regions (aHR 8.26, 95% CI 5.43–12.56; all p < 0.01). Several treatment-related factors were independently associated with improved retention. Optimal dosing (aHR 0.74, 95% CI 0.69–0.78; p < 0.01), unsupervised dosing (aHR 0.71, 95% CI 0.64–0.78; p < 0.01), and longer (180+ days) pre-war OAMT episode duration (aHR 0.65, 95% CI 0.54–0.77; p < 0.01) were associated with lower discontinuation risk, whereas participants with interrupted prior OAMT episodes were more likely to discontinue treatment (aHR 1.39, 95% CI 1.29–1.50; all p < 0.01). Medication type was not independently associated with discontinuation.

**Table 2:** Multivariable frailty-adjusted Cox proportional hazards model of factors associated with opioid agonist maintenance treatment (OAMT) discontinuation in Ukraine (N = 17,211).

Characteristic	Category	aHR	Lower 95% CI	Upper 95% CI	p-value
Displacement status^a^	Low-intensity war local	Ref.			
	High-intensity war local	1.74	1.4	2.15	<0.01
	Frontline local	3.09	2.8	3.42	<0.01
	Low-intensity war IDP	3.55	2.92	4.32	<0.01
	High-intensity war IDP	5.9	4.52	7.69	<0.01
	Frontline IDP	8.26	5.43	12.56	<0.01
Medication^a^	Methadone (MET)	Ref.			
	Buprenorphine (BUP)	1.06	0.97	1.16	0.24
Dosage^a^, mg	Suboptimal	Ref.			
	Optimal	0.74	0.69	0.78	<0.01
Dosing strategy^a^	Daily supervised	Ref.			
	Unsupervised	0.71	0.64	0.78	<0.01
Episode type	First OAMT episode	Ref.			
	Not first OAMT episode	1.39	1.29	1.5	<0.01
Baseline OAMT episode duration	≤30 days	Ref.			
	31–180 days	0.88	0.73	1.07	0.19
	>180 days	0.65	0.54	0.77	<0.01
Age, years	<34	Ref.			
	≥34	0.97	0.91	1.05	0.45
Sex	Male	Ref.			
	Female	1.2	1.11	1.3	<0.01
HIV status	HIV-negative	Ref.			
	HIV-positive	1.05	0.98	1.12	0.16
Drug injection duration, years	≥13	Ref.			
	<13	0.98	0.91	1.06	0.63
Pre-War population quartile^a^	Q1 (0.9–1.0 million)	Ref.			
	Q2 (1.1–1.2 million)	1.18	1.04	1.35	0.01
	Q3 (1.4–2.1 million)	2.26	2.01	2.54	<0.01
	Q4 (2.4–4.1 million)	1.86	1.65	2.09	<0.01
Pre-War GRDP per capita quartile^a^	Q1 (€0.5 k–1.7 k)	Ref.			
	Q2 (€1.7 k–2.1 k)	0.3	0.26	0.34	<0.01
	Q3 (€2.2 k–2.7 k)	0.25	0.22	0.28	<0.01
	Q4 (€3.2 k–8.4 k)	0.15	0.12	0.19	<0.01
Pre-War region OAMT performance category^a^	High acquisition/low dropout	Ref.			
	Mid-level acquisition/high dropout	1.15	1.02	1.30	0.03
	Low acquisition/mid-level dropout	1.36	1.21	1.54	<0.01

Legend: IDP: Internally Displaced Persons.

a Time-dependent covariates.

Among baseline demographic characteristics, women were more likely than men to discontinue treatment (aHR 1.20, 95% CI 1.11–1.30; p < 0.01), whereas HIV status, age, and duration of drug injection were not independently associated with discontinuation in adjusted models. Regional structural characteristics were also associated with retention. Participants in more populous oblasts had higher discontinuation risk, whereas higher pre-war regional GRDP per capita was strongly protective. Compared with regions characterised by high patient acquisition and low dropout before the war, regions with lower pre-war acquisition and higher pre-war dropout demonstrated higher discontinuation risk during wartime.

In stratified frailty-adjusted analyses (Table 3), the effects of displacement and treatment characteristics varied substantially across regional contexts. Displacement into high-intensity and frontline regions was consistently associated with elevated discontinuation risk across strata, whereas displacement from frontline into low-intensity regions was associated with lower discontinuation risk relative to remaining locally within frontline regions (aHR 0.52, 95% CI 0.42–0.64; p < 0.01). Optimal dosing and unsupervised dosing remained protective across most strata, although the protective effect of optimal dosing was attenuated in high-intensity regions. Buprenorphine use was associated with higher discontinuation risk only in high-intensity regions (aHR 1.45, 95% CI 1.21–1.74; p < 0.001). Pre-war OAMT episode discontinuation and female sex were consistently associated with higher discontinuation risk across strata, whereas HIV status predicted discontinuation only in frontline regions (aHR 1.10, 95% CI 1.01–1.19; p = 0.02).

**Table 3:** Stratified frailty-adjusted Cox models by region clusters.

Characteristic	Low-intensity war				High-intensity war				Frontline			
	aHR	95% CI		p	aHR	95% CI		p	aHR	95% CI		p
Strata-specific local	Ref.				Ref.				Ref.			
Low-intensity war IDP	1.82	0.44	7.5	0.41	3.04	1.51	6.13	<0.01	0.52	0.42	0.64	<0.01
High-intensity war IDP	4.39	1.87	10.32	<0.01	4.32	2.12	8.82	<0.01	0.98	0.8	1.2	0.87
Frontline IDP	4.34	1.3	14.52	0.02	3.76	0.72	19.57	0.12	3.12	1.98	4.92	<0.01
Methadone	Ref.				Ref.				Ref.			
Buprenorphine	0.98	0.78	1.22	0.84	1.45	1.21	1.74	<0.01	1.09	0.97	1.22	0.15
Suboptimal dose	Ref.				Ref.				Ref.			
Optimal dose	0.73	0.62	0.86	<0.01	0.93	0.82	1.05	0.24	0.69	0.63	0.74	<0.01
Daily supervised	Ref.				Ref.				Ref.			
Unsupervised dosing	0.48	0.38	0.6	<0.01	0.58	0.48	0.69	<0.01	0.84	0.74	0.96	0.01
1st OAMT episode	Ref.				Ref.				Ref.			
2nd + OAMT episode	1.73	1.43	2.09	<0.01	1.57	1.35	1.83	<0.01	1.23	1.12	1.36	<0.01
≤30 days on OAMT	Ref.				Ref.				Ref.			
31–180 days on OAMT	0.73	0.45	1.18	0.2	0.81	0.57	1.14	0.22	1.01	0.78	1.29	0.97
>180 days on OAMT	0.34	0.22	0.53	<0.01	0.43	0.31	0.59	<0.01	0.95	0.76	1.2	0.67
<34 years old	Ref.				Ref.				Ref.			
≥34 years old	0.95	0.79	1.13	0.57	0.92	0.8	1.06	0.27	1	0.91	1.09	0.94
Male	Ref.				Ref.				Ref.			
Female	1.49	1.18	1.88	<0.01	1.27	1.09	1.47	<0.01	1.17	1.06	1.3	<0.01
HIV-negative	Ref.				Ref.				Ref.			
HIV-positive	0.85	0.71	1.01	0.07	0.97	0.86	1.1	0.63	1.1	1.01	1.19	0.02
≥13 years of IDU	Ref.				Ref.				Ref.			
<13 years of IDU	0.92	0.77	1.11	0.39	0.97	0.83	1.12	0.64	1.07	0.98	1.17	0.15
Regional population Q1	Ref.				Ref.				Ref.			
Regional population Q2-Q4	1.26	1	1.58	0.05	0.43	0.1	1.85	0.26	1.65	1.48	1.85	<0.01
GRDP Q1	Ref.				Ref.				Ref.			
GRDP Q2-Q4	0.95	0.69	1.31	0.75	1.22	0.21	6.99	0.82	0.25	0.23	0.28	<0.01
High acquisition/low DO regions	Ref.				Ref.				Ref.			
Mid-level acquisition/high DO regions	1.43	1.1	1.85	<0.01	1.23	1.05	1.44	0.01	0.38	0.32	0.44	<0.01
Low acquisition/mid-level DO regions	1.05	0.79	1.41	0.72	0.93	0.79	1.09	0.38	0.63	0.53	0.74	<0.01

Legend: IDP: Internally Displaced Persons, DO: dropout, Q: quartile; OAMT: opioid agonist maintenance treatment.

## Discussion

In this national prospective cohort study of people with OUD receiving OAMT in Ukraine, treatment continuity during wartime was strongly shaped by displacement and regional war intensity. Participants residing in frontline regions and those experiencing internal displacement faced substantially higher risk of treatment discontinuation, whereas optimised dosing and unsupervised dosing were independently associated with improved retention. Despite prolonged armed conflict and large-scale population displacement, overall OAMT retention remained relatively high (70%) during follow-up, and national program capacity expanded by 17% during the war period.^13^ This expansion occurred despite substantial wartime population displacement and increased demand for OAMT services, including enrolment of internally displaced patients, transfers from disrupted private clinics, and initiation of treatment among individuals whose access to non-prescribed opioids was interrupted during the war.^10^ Although the study design does not permit direct evaluation of the organisational mechanisms underlying these patterns, the findings identify several regional and treatment-related characteristics associated with treatment continuity during wartime and suggest both the vulnerability and adaptability of OAMT systems during humanitarian crises.

Large-scale interruptions in OAMT supply were generally not observed outside occupied territories, where prolonged disruption of methadone access was documented, particularly in parts of Kherson oblast.^10^ Earlier qualitative work from Ukraine suggests that wartime disruptions primarily affected transportation logistics, regional medication distribution, and local service delivery rather than national medication availability.^10^ In response, regions rapidly redistributed medication supplies, expanded unsupervised dosing, and implemented flexible inter-regional transfer mechanisms to maintain continuity of care.^6^ Patients already enrolled in OAMT were generally prioritised during the wartime response, and we did not observe systematic reductions in dosing levels during follow-up.^10^

Ukraine's ability to sustain and expand OAMT enrolment during a period of extreme system disruption underscores both the resilience of OAMT delivery and the importance of sustained implementation and policy investments before the full-scale invasion. Earlier patient-led advocacy, sustained support from the U.S. President's Emergency Plan for AIDS Relief (PEPFAR) and the Global Fund, and national policy reforms helped transition Ukraine's OAMT system toward a more flexible, patient-centred model of care.^11^^,^^12^^,^^22^ Following the earlier invasion in eastern Ukraine in 2014 and later during the COVID-19 pandemic, the OAMT program developed mechanisms for patient transfer, re-enrolment, expanded unsupervised dosing, and continuity of care during disruption.^10^^,^^12^^,^^23^ These earlier reforms and investments, including NIH-supported NIATx initiatives to improve clinic efficiency, retention, and quality of care, likely strengthened organisational readiness before the full-scale invasion.^18^^,^^19^ Parallel integration of methadone services into primary care and expanding flexible delivery models further enhanced continuity of care under conditions of stress.^24^ During the full-scale war in 2022, collaboration between public and private sites, redistribution of medication stocks, and flexible transfer procedures helped maintain continuity of care despite severe disruption to transportation, supply chains, and clinic access.^9^^,^^25^

Given the high prevalence of injection drug use among people with OUD in Ukraine, sustaining OAMT delivery is essential for HIV prevention.^26^ Previous research has consistently shown that OAMT is both effective and cost-effective for HIV prevention and care across Eastern Europe and Central Asia. Modelling studies specific to Ukraine demonstrate that scaling up OAMT reduces HIV incidence among PWID and remains highly cost-effective even within constrained health budgets.^3^ Registry-based analyses have further demonstrated that OAMT supports engagement at all stages of the HIV care cascade, including higher uptake of testing, antiretroviral therapy initiation, and retention in treatment.^27^ Beyond treatment outcomes, molecular epidemiology has provided compelling evidence that war itself drives HIV spread in Ukraine: viral phylogenetic analyses showed that the 2014 war in the east facilitated the movement of HIV lineages across the country, with displaced PWID and their sexual and injecting networks playing a central role in onward transmission.^28^ Our findings extend this evidence to the full-scale wartime context, underscoring the urgent need to sustain OAMT for PWID, particularly in regions affected by high war intensity. Although HIV status was not independently associated with discontinuation overall, sustaining OAMT continuity remains critically important for preserving HIV prevention and treatment infrastructure during wartime disruption. Failure to sustain OAMT continuity during war risks reversing hard-won gains in HIV prevention and treatment. As highlighted in the recent application of the Big Events framework, the current war risks unleashing a broader HIV epidemic in Ukraine if harm reduction and OAMT programs are not protected and scaled up.^29^

Internal displacement emerged as a major driver of treatment discontinuation, particularly among individuals displaced into high-intensity and frontline regions. This pattern mirrors evidence from humanitarian settings globally, where disruption of OAMT among refugees and internally displaced persons has been linked to overdose and interruptions in HIV care.^16^ Displacement amplifies the mismatch between patient needs and receiving-site capacity while exposing regulatory and administrative barriers that are often less visible in peacetime.^15^ These findings are also important for HIV control because displacement during war can reconfigure transmission networks, and war-related population movement has previously been implicated in HIV spread in Ukraine.^28^ Further, studies from diverse humanitarian settings have shown that community-based OAMT is feasible during crises but fragile in the face of restrictive policies and donor dependency.^14^^,^^30^ Protecting OAMT continuity for displaced populations therefore serves a dual purpose: reducing overdose and withdrawal harms while preserving HIV prevention in disrupted risk environments.^27^^,^^31^ The comparatively lower discontinuation observed among individuals displaced from frontline regions may reflect a selection effect whereby those able to relocate from areas of active combat were more likely to successfully engage with receiving OAMT programs.

Beyond displacement *per se*, the intensity of war exposure, particularly in frontline and high-intensity regions, was strongly associated with OAMT discontinuation. High-intensity exposure probably reflects multiple interacting pathways, including direct threats to safety, intermittent closure of services due to shelling or security restrictions, disrupted transportation corridors, migration and displacement of both patients and healthcare workers, and degradation of supply chains.^9^ Displacement and redeployment of healthcare personnel likely further contributed to regional variability in OAMT continuity, particularly in frontline and occupied regions where workforce shortages and healthcare system fragmentation may have reduced local treatment capacity.^9^ Global experience from other conflicts reinforces that attacks on health facilities and the erosion of health system functioning are predictable features of modern warfare, with downstream effects on continuity of care for chronic conditions, including substance use disorders and HIV.^32^ Qualitative work from Ukraine further suggests that war-related disruptions increase risks of withdrawal, re-emergence of use, and diversion, while simultaneously increasing urgency to secure medication when routines collapse.^33^ These findings support incorporating war-intensity metrics into emergency preparedness planning for OAMT, including pre-positioning medication stocks, building redundancy across sites, and formalising patient transfer pathways that do not depend on stable infrastructure.

In addition to geography, dosing and the availability of unsupervised dosing were strongly protective against discontinuation, which has important policy relevance during emergency response. This aligns with a robust pre-war and pandemic-era evidence base showing that adequate dosing improves retention, and that unsupervised dosing, when implemented with clinical judgement and safeguards, can support continuity, reduce the burden of daily attendance, and preserve engagement when mobility is constrained.^18^ Over the study period, the proportion of patients receiving suboptimal doses also declined nationally (Supplemental Figure S1), while optimal dosing remained independently associated with lower discontinuation risk. Although the observational design does not permit causal inference regarding underlying mechanisms, these findings are consistent with prior evidence linking adequate methadone and buprenorphine dosing to improved treatment retention and support continued emphasis on evidence-based dosing practices during periods of war and healthcare disruption. Similar lessons have been documented in non-war disasters, such as Hurricane Sandy, where regulatory flexibility and pragmatic delivery adaptations were central to maintaining access to methadone and buprenorphine.^34^ International guidance has long recognised methadone and buprenorphine as core evidence-based treatments for OUD and key strategies for HIV prevention, while contemporary guidance continues to emphasise OAMT as foundational for overdose prevention.^35^ In war settings, the practical conclusion is that protocols for rapidly expanding unsupervised dosing, paired with patient-centred risk stratification and clear diversion mitigation plans, should be treated as standard emergency preparedness, not exceptional policy waivers.

The divergent retention patterns observed by medication type across regions suggest that treatment continuity was shaped not only by war exposure but also by local economic context, treatment financing, and medication access pathways. After controlling for regional economic characteristics, the association between medication type and retention became more apparent and was consistent with previous studies showing lower retention among patients receiving buprenorphine compared with methadone.^36^ These findings suggest that maintaining OAMT continuity during war requires not only preserving medication supply chains, but also reducing reliance on privately financed treatment pathways, particularly when buprenorphine is obtained through out-of-pocket payment, which may become unstable during periods of displacement, economic disruption, and healthcare fragmentation.^9^^,^^18^ Expanding publicly supported buprenorphine access, standardising emergency dispensing policies across regions, and strengthening integrated public-sector delivery systems may improve retention during humanitarian crises, particularly in urban areas where private treatment markets play a larger role.

Gender-related barriers to OAMT retention are well-documented and were reflected in our findings, with women experiencing higher discontinuation risk than men.^37^ Although men in the general Ukrainian population were subject to wartime mobility restrictions, patients receiving OAMT were exempt from military conscription under Order No. 402 of the Ministry of Health of Ukraine, which permitted them to leave the country, suggesting that the observed sex differences in retention were unlikely to be explained solely by legal restrictions on male mobility. Notably, this pattern has not been observed in pre-war OAMT studies from Ukraine,^11^^,^^12^^,^^18^ suggesting that the sex differences identified here may reflect wartime disruption and displacement dynamics. Women may have been more likely to relocate internationally during the war, including for caregiving and child safety reasons, while simultaneously facing gender-specific barriers to treatment continuity, including stigma, trauma burden, childcare responsibilities, and heightened vulnerability to exploitation and violence during displacement.^37^ On the other hand, men may have had additional incentives to remain engaged in OAMT because of conscription exemption. Nevertheless, these findings support the need for gender-responsive OAMT models during humanitarian crises, including childcare-compatible dosing schedules, proactive outreach for displaced women, and integrated referral pathways for gender-based violence and social support services.^37^

Several limitations of this study should be acknowledged. First, although inter-oblast transfers recorded in SyRex provide a useful proxy for displacement, they likely underestimate the true scale of population mobility during the war. Some displaced individuals may have discontinued OAMT without formally transferring to another site, and we were unable to systematically capture inter-country relocations, which were frequent and may have resulted in treatment discontinuation outside Ukraine's national system. Second, exact dates of changes in OAMT dose and dispensing modality were not available in SyRex, as these variables are updated through standardised quarterly reporting procedures rather than real-time clinical records, which may have introduced temporal misalignment between treatment-related variables and rapidly fluctuating wartime conditions. Third, regional war intensity classifications relied on administrative indicators that may not fully capture local security conditions. Fourth, SyRex does not include key social determinants of health or comprehensive psychiatric comorbidity data that may have influenced treatment continuity. Finally, although the analyses incorporated baseline regional program performance indicators derived from a prior national implementation study, the registry structure does not fully capture the organisational, operational, or patient-level mechanisms underlying regional differences in treatment continuity. Accordingly, the observed associations should be interpreted primarily as epidemiologic patterns rather than direct evidence of specific implementation processes or causal pathways. Despite these limitations, SyRex represents one of the most comprehensive national OAMT registries globally and enabled longitudinal, individual-level analyses of displacement, treatment continuity, and program adaptation during active war. Moreover, the use of frailty-adjusted survival models accounted for repeated treatment episodes within individuals, and modelling regional level and clinical characteristic variables as time-dependent covariates allowed us to capture dynamic patterns of patient mobility. The absence of missing data further strengthens the robustness and internal validity of our estimates.

### Conclusion

Our findings suggest that sustaining OAMT during war through modifiable clinical and programmatic aspects of OAMT delivery is feasible and may play an important role in reducing risks associated with untreated OUD during humanitarian crises. In Ukraine, flexible treatment delivery models, including rapid expansion of unsupervised dosing, simplified interregional transfer mechanisms, and adaptive collaborative program coordination, helped preserve continuity of care despite large-scale displacement, active combat, and severe healthcare disruption. These adaptations could prevent substantial individual and population-level harms during one of the largest humanitarian crises in modern Europe. The importance of maintaining OAMT during war extends far beyond addiction treatment alone. OAMT remains one of the most effective HIV prevention interventions for PWID and is critical for sustaining engagement across the HIV treatment cascade.^27^^,^^31^ Historical experience from Ukraine also illustrates what happens when OAMT is withdrawn in occupied territories, with documented harms following the cessation of methadone in Crimea.^23^ Conflict preparedness for OAMT should therefore be positioned alongside other essential continuity strategies, with a focus on simplified transfer mechanisms, rapid expansion of unsupervised dosing, redundant medication supply chains, and cross-jurisdiction coordination for displaced populations. In doing so, health systems can transform OAMT from a vulnerable point of failure into a stabilising platform that protects both individual wellbeing and population-level HIV prevention during war.

## Contributors

RI and FLA conceived the study. RI, LMM, DJB, EM, BA, SOF, and FLA contributed to the study design and methodology. RI and EM led data curation, formal analyses, visualisation, and project administration. AM, TF, MF, and IK contributed to data curation, investigation, and project administration. ZI provided resources and supervision. LMM and FLA acquired funding. RI drafted the original manuscript. All authors contributed to interpretation of the findings, critically reviewed and revised the manuscript, approved the final version for publication, and accept responsibility for the decision to submit the manuscript. RI accessed and verified the underlying data.

## Data sharing statement

The data analysed in this study were obtained from the Ukrainian national opioid agonist maintenance treatment registry and are not publicly available because of legal and ethical restrictions. De-identified data may be made available from the Ukrainian Alliance for Public Health upon reasonable request and with appropriate institutional approvals and data use agreements, subject to applicable Ukrainian regulations.

## Editor note

The Lancet Group takes a neutral position with respect to territorial claims in published maps and institutional affiliations.

## Declaration of interests

All authors declare no competing interests.
