# Supplementary material for: War-related displacement, conflict intensity, and treatment delivery factors in opioid agonist maintenance treatment discontinuation following Russia's invasion of Ukraine: a national cohort study
Source: Lancet Reg Health Eur. 2026 Aug 26;69:101831. doi: 10.1016/j.lanepe.2026.101831 (PMC13527472; doi:10.1016/j.lanepe.2026.101831)
Supplement: Supplementary file 1 [file mmc1.docx]

# Supplementary Appendix

War-Related Displacement and Opioid Agonist Maintenance Therapy Discontinuation Following Russia's Full-Scale Invasion of Ukraine: A National Longitudinal Cohort Study

**Contents**

| **Section** | **Page** |
| --- | --- |
| **Part I. Supplementary Descriptive Analyses** | 2 |
| Supplementary Table S1. Monthly Opioid Agonist Therapy Retention and Mobility Indicators Across Regional Conflict Clusters, February 23, 2022–January 1, 2024 | 2 |
| Supplementary Figure S1. Quarterly Trends in Opioid Agonist Therapy Retention by Dosing and Dispensing Strategy, Ukraine, February 2022–January 2024 | 3 |
| **Part II. K-means Clustering Methods and Results** | 4 |
| Table A1. Region-level Indicators with Assigned Cluster | 5 |
| Figure A1. Gap Statistic for Selection of the Optimal Number of War Exposure Clusters Across Ukrainian Regions | 6 |
| Table A2. K-means Cluster Membership and Quality Metrics (k = 3) | 6 |
| Figure A2. Visualization of K-means War Exposure Clusters Across Ukrainian Regions | 7 |
| **Part III. Pre-war Cross-regional Migration of OAMT Patients** | 8 |
| **Part IV. Proportional Hazards Assumption Assessment** | 8 |
| Table A3. Schoenfeld Residual Tests for the Final Cox Regression Model | 8 |
| Figure A3. Scaled Schoenfeld Residual Plots for the Final Cox Regression Model | 9 |
| **Part V. Pre-war Regional OAMT Programme Performance Classification** | 10 |
| Table A4. Ukraine's Regions and Corresponding NIATx Measures | 10 |
| Figure A4. Acquisition Rate by Month by Cluster (2017–2021) | 11 |
| Figure A5. Dropout Rate by Month by Cluster (2017–2021) | 11 |
| Supplementary References | 12 |
| **Part VI. Sensitivity Analysis** | 13 |
| Supplementary Table S2. Sensitivity Analysis Using a 30-Day Definition of OAMT Dropout | 13 |
|  |  |

# I. Supplementary Descriptive Analyses

**Supplementary Table S1.** Monthly Opioid Agonist Therapy Retention and Mobility Indicators Across Regional Conflict Clusters, February 23^rd^, 2022 – January 1^st^, 2024

| TIME | Remote Region Local | | Remote Region IDP | | TargetED Region Local | | TargetED Region IDP | | Frontilne Region Local | | Frontline Region IDP | | Interim Dropout | | Permanent Dropout | | Monthly Retention | |
| --- | --- | --- | --- | --- | --- | --- | --- | --- | --- | --- | --- | --- | --- | --- | --- | --- | --- | --- |
|  | **N** | **%*** | **N** | **%*** | **N** | **%*** | **N** | **%*** | **N** | **%*** | **N** | **%*** | **N** | **%**** | **N** | **%***** | **N** | **%** |
| 2022-02 | 4631 | 100 | 0 | 0 | 6998 | 100 | 0 | 0 | 5582 | 100 | 0 | 0 | 0 | 0 | 0 | 0 | 17211 | 100.34 |
| 2022-03 | 4599 | 99.31 | 0 | 0 | 6976 | 99.69 | 0 | 0 | 5499 | 98.51 | 0 | 0 | 69 | 0.4 | 68 | 0.4 | 17074 | 99.2 |
| 2022-04 | 4500 | 97.17 | 87 | 1.88 | 6764 | 96.66 | 59 | 0.84 | 4811 | 86.19 | 12 | 0.21 | 322 | 1.87 | 710 | 4.13 | 16233 | 94.32 |
| 2022-05 | 4444 | 95.96 | 214 | 4.62 | 6639 | 94.87 | 198 | 2.83 | 4329 | 77.55 | 15 | 0.27 | 316 | 1.84 | 1110 | 6.45 | 15839 | 92.03 |
| 2022-06 | 4381 | 94.6 | 237 | 5.12 | 6580 | 94.03 | 230 | 3.29 | 4072 | 72.95 | 21 | 0.38 | 291 | 1.69 | 1453 | 8.44 | 15521 | 90.18 |
| 2022-07 | 4338 | 93.67 | 230 | 4.97 | 6504 | 92.94 | 228 | 3.26 | 4026 | 72.12 | 21 | 0.38 | 287 | 1.67 | 1631 | 9.48 | 15347 | 89.17 |
| 2022-08 | 4285 | 92.53 | 225 | 4.86 | 6444 | 92.08 | 231 | 3.3 | 3537 | 63.36 | 21 | 0.38 | 457 | 2.66 | 2065 | 12 | 14743 | 85.66 |
| 2022-09 | 4244 | 91.64 | 226 | 4.88 | 6368 | 91 | 233 | 3.33 | 3381 | 60.57 | 26 | 0.47 | 467 | 2.71 | 2320 | 13.48 | 14478 | 84.12 |
| 2022-10 | 4206 | 90.82 | 223 | 4.82 | 6302 | 90.05 | 246 | 3.52 | 3281 | 58.78 | 25 | 0.45 | 416 | 2.42 | 2566 | 14.91 | 14283 | 82.99 |
| 2022-11 | 4173 | 90.11 | 212 | 4.58 | 6219 | 88.87 | 254 | 3.63 | 3209 | 57.49 | 21 | 0.38 | 405 | 2.35 | 2772 | 16.11 | 14088 | 81.85 |
| 2022-12 | 4116 | 88.88 | 203 | 4.38 | 6159 | 88.01 | 259 | 3.7 | 3176 | 56.9 | 32 | 0.57 | 392 | 2.28 | 2928 | 17.02 | 13945 | 81.02 |
| 2023-01 | 4079 | 88.08 | 204 | 4.41 | 6105 | 87.24 | 252 | 3.6 | 3193 | 57.2 | 17 | 0.3 | 278 | 1.62 | 3137 | 18.23 | 13850 | 80.47 |
| 2023-02 | 4028 | 86.98 | 207 | 4.47 | 6051 | 86.47 | 246 | 3.52 | 3161 | 56.63 | 15 | 0.27 | 254 | 1.48 | 3303 | 19.2 | 13708 | 79.65 |
| 2023-03 | 3987 | 86.09 | 204 | 4.41 | 5964 | 85.22 | 243 | 3.47 | 3119 | 55.88 | 17 | 0.3 | 261 | 1.52 | 3470 | 20.17 | 13534 | 78.64 |
| 2023-04 | 3930 | 84.86 | 200 | 4.32 | 5889 | 84.15 | 243 | 3.47 | 3084 | 55.25 | 16 | 0.29 | 255 | 1.48 | 3648 | 21.2 | 13362 | 77.64 |
| 2023-05 | 3886 | 83.91 | 199 | 4.3 | 5830 | 83.31 | 238 | 3.4 | 3049 | 54.62 | 17 | 0.3 | 234 | 1.36 | 3812 | 22.15 | 13219 | 76.81 |
| 2023-06 | 3849 | 83.11 | 191 | 4.12 | 5767 | 82.41 | 246 | 3.52 | 3035 | 54.37 | 16 | 0.29 | 214 | 1.24 | 3947 | 22.94 | 13104 | 76.14 |
| 2023-07 | 3816 | 82.4 | 192 | 4.15 | 5726 | 81.82 | 239 | 3.42 | 3020 | 54.1 | 18 | 0.32 | 197 | 1.14 | 4057 | 23.58 | 13011 | 75.6 |
| 2023-08 | 3774 | 81.49 | 187 | 4.04 | 5667 | 80.98 | 228 | 3.26 | 2995 | 53.65 | 22 | 0.39 | 172 | 1 | 4220 | 24.52 | 12873 | 74.8 |
| 2023-09 | 3712 | 80.16 | 189 | 4.08 | 5604 | 80.08 | 223 | 3.19 | 2965 | 53.12 | 23 | 0.41 | 142 | 0.83 | 4407 | 25.61 | 12716 | 73.88 |
| 2023-10 | 3693 | 79.75 | 188 | 4.06 | 5549 | 79.29 | 230 | 3.29 | 2954 | 52.92 | 23 | 0.41 | 102 | 0.59 | 4526 | 26.3 | 12637 | 73.42 |
| 2023-11 | 3628 | 78.34 | 189 | 4.08 | 5423 | 77.49 | 230 | 3.29 | 2927 | 52.44 | 23 | 0.41 | 90 | 0.52 | 4755 | 27.63 | 12420 | 72.16 |
| 2023-12 | 3581 | 77.33 | 190 | 4.1 | 5380 | 76.88 | 226 | 3.23 | 2908 | 52.1 | 23 | 0.41 | 36 | 0.21 | 4921 | 28.6 | 12308 | 71.51 |
| 2024-01 | 3549 | 76.64 | 188 | 4.06 | 5344 | 76.36 | 229 | 3.27 | 2890 | 51.77 | 25 | 0.45 | 0 | 0 | 5040 | 29.29 | 12225 | 71.03 |

**Legend:**

**IDP** – Internally Displaced People.

***** – percentage of internally displaced OAT patients relative to the total number of OAT patients within each regional conflict cluster for the corresponding month.

****** – the proportion of patients temporarily interrupting treatment but returning within the study period—calculated relative to the total number of patients enrolled at the beginning of each month.

******* –indicates the proportion of patients who discontinued treatment without documented return—calculated relative to the total number of patients at the beginning of study period.

**Supplemental Figure 1.** Quarterly Trends in Opioid Agonist Therapy (OAT) Retention by Dosing and Dispensing Strategy, Ukraine, February 2022–January 2024

# II. K-means Clustering Methods and Results

War exposure clusters were developed using six regional indicators: number of air raids, explosions, artillery attacks, percentage of internally displaced persons, occupation days, and frontline days (see Table A1). Because several variables were highly skewed, air raids, explosions, artillery attacks, occupation days, IPD proportion, and frontline days were log-transformed using log(x+1), and all variables were standardized prior to clustering. K-means clustering with 50 random starts was then applied to classify regions into groups with similar war exposure profiles.

To evaluate candidate clustering solutions, models ranging from 1 to 6 clusters were assessed using the gap statistic (Figure A1), silhouette width, Calinski-Harabasz index, Davies-Bouldin index, explained variance, and epidemiologic interpretability. The gap statistic increased progressively from 0.021 for 1 cluster to 0.170 for 2 clusters and 0.215 for 3 clusters, with smaller gains thereafter (0.245 for 4 clusters, 0.290 for 5 clusters, and 0.314 for 6 clusters). Although the formal firstSEmax criterion suggested a 5-cluster solution, improvements beyond the 3-cluster solution were incremental relative to the loss of interpretability and the emergence of smaller regional groupings. Consistent with this finding, the elbow plot demonstrated a marked reduction in within-cluster variability between 1 and 3 clusters, followed by substantial flattening of the curve thereafter, supporting the selection of a 3-cluster solution as the most parsimonious and epidemiologically meaningful classification. The 3-cluster solution also preserved sufficient regional sample size within each exposure category to support stable estimation in subsequent time-dependent survival analyses.

Average silhouette widths were also examined across candidate solutions. The 2-cluster solution produced the highest silhouette width (0.503), indicating the strongest statistical separation between groups. However, the 3-cluster solution retained moderate separation (overall silhouette width ≈0.44) while providing greater substantive differentiation between Low-intensity, High-intensity, and Frontline war exposure regions. Silhouette widths progressively declined for solutions with 4 or more clusters, indicating increasing overlap and reduced cluster cohesion. The final 3-cluster solution demonstrated acceptable cluster validity and internal consistency, with a Calinski-Harabasz index of 24.87, a Davies-Bouldin index of 0.97, and 70.3% of total variability explained by between-cluster differences.

Table A2 summarizes cluster membership and cluster quality metrics for the final 3-cluster solution. The Low-intensity cluster represented the largest and most internally cohesive grouping, containing 50.0% of regions and demonstrating the highest average silhouette width (0.547), together with the lowest within-cluster sum of squares (10.3), indicating good cluster compactness and separation. The Frontline cluster included 29.2% of regions and demonstrated moderate cohesion (silhouette width = 0.404). The High-intensity cluster contained 20.8% of regions and showed lower silhouette width (0.238), suggesting greater heterogeneity and overlap with neighboring exposure profiles, likely reflecting the complex and variable nature of heavily war-affected non-frontline regions.

ANOVA comparisons demonstrated statistically significant between-cluster differences for air raids (p<0.001), explosions (p=0.002), IDP burden (p<0.001), occupation days (p<0.001), and frontline exposure duration (p<0.001), supporting the construct validity of the final clustering solution. Differences in artillery activity did not reach statistical significance (p=0.166).

The final clusters were ordered by overall exposure intensity and labeled as Low-intensity, High-intensity, and Frontline. The Low-intensity cluster included 12 regions and was characterized by relatively low levels of air raids, explosions, artillery activity, displacement, occupation, and frontline exposure. The High-intensity cluster included 5 regions characterized by elevated artillery activity, substantial conflict intensity, and moderate air raid exposure, but comparatively lower frontline and occupation burden than the Frontline cluster. The Frontline cluster included 7 regions and had the highest levels of air raids and explosions, together with substantial frontline and occupation exposure. Figure A2 presents the visual distribution of regions across the final cluster solution using the first two principal component dimensions, which together explained approximately 86.0% of the overall variance (Dim1: 66.7%; Dim2: 20.1%). Principal component analysis was used solely for visualization of cluster structure and was not used to derive the clusters.

## Table A1. Region-level Indicators with Assigned Cluster

| **Region** | **Air raids** | **Explosions** | **Artillery** | **IDP %** | **Occupation days** | **Frontline days** | **Cluster** | **Cluster label** |
| --- | --- | --- | --- | --- | --- | --- | --- | --- |
| Cherkasy | 1,094 | 39 | 0 | 4.5 | 0 | 0 | 1 | Low intensity |
| Chernivtsi | 423 | 1 | 0 | 2.5 | 0 | 0 | 1 | Low intensity |
| Ivano-Frankivsk | 444 | 5 | 0 | 2.5 | 0 | 0 | 1 | Low intensity |
| Khmelnytskyi | 550 | 101 | 0 | 2.5 | 0 | 0 | 1 | Low intensity |
| Kirovohrad | 1,457 | 44 | 1 | 2.5 | 0 | 0 | 1 | Low intensity |
| Lviv | 446 | 58 | 0 | 4.5 | 0 | 0 | 1 | Low intensity |
| Rivne | 530 | 15 | 0 | 0.5 | 0 | 0 | 1 | Low intensity |
| Ternopil | 513 | 10 | 0 | 2.5 | 0 | 0 | 1 | Low intensity |
| Vinnytsia | 830 | 63 | 0 | 2.5 | 0 | 0 | 1 | Low intensity |
| Volyn | 536 | 15 | 0 | 0.5 | 0 | 0 | 1 | Low intensity |
| Zakarpattia | 386 | 1 | 0 | 2.5 | 0 | 0 | 1 | Low intensity |
| Zhytomyr | 817 | 46 | 3 | 2.5 | 0 | 0 | 1 | Low intensity |
| Kyiv City | 945 | 281 | 0 | 8.0 | 38 | 38 | 2 | High intensity |
| Kyiv Region | 835 | 281 | 0 | 8.0 | 38 | 38 | 2 | High intensity |
| Odesa | 1,014 | 260 | 0 | 8.0 | 0 | 0 | 2 | High intensity |
| Poltava | 1,674 | 83 | 0 | 8.0 | 0 | 0 | 2 | High intensity |
| Dnipropetrovsk | 2,624 | 460 | 8,579 | 12.5 | 0 | 0 | 2 | High intensity |
| Kharkiv | 3,211 | 767 | 2,388 | 12.5 | 677 | 677 | 3 | Frontline |
| Donetsk | 3,585 | 1,176 | 696 | 0.0 | 677 | 677 | 3 | Frontline |
| Kherson | 1,544 | 2,885 | 829 | 0.0 | 677 | 677 | 3 | Frontline |
| Mykolaiv | 1,735 | 296 | 862 | 4.5 | 261 | 261 | 3 | Frontline |
| Chernihiv | 1,014 | 39 | 7 | 2.5 | 43 | 677 | 3 | Frontline |
| Sumy | 1,454 | 1,200 | 2,354 | 2.5 | 44 | 677 | 3 | Frontline |
| Zaporizhzhia | 3,250 | 607 | 354 | 0.0 | 675 | 677 | 3 | Frontline |

## Figure A1. Gap Statistic for Selection of the Optimal Number of War Exposure Clusters Across Ukrainian Regions

## Table A2. K-means Cluster Membership and Quality Metrics (k = 3)

| **Cluster** | **N Regions** | **Percent of Regions** | **Within-Cluster SS** | **Average Silhouette Width** |
| --- | --- | --- | --- | --- |
| 1 | 7 | 29.2 | 18.5 | 0.404 |
| 2 | 12 | 50.0 | 10.3 | 0.561 |
| 3 | 5 | 20.8 | 13.0 | 0.238 |

## Figure A2. Visualization of K-means War Exposure Clusters Across Ukrainian Regions

III. **Pre-pandemic cross regional migration of OAMT patients**

IV. **Proportional Hazar Assumption Test Results and Interpretation**

**Table A3. Variable definitions and Schoenfeld residual tests for the proportional hazard assumption in the final Cox regression model.**

| **Variable** | **Description** | **χ²** | **df** | **p-value** | **Variables Type** |
| --- | --- | --- | --- | --- | --- |
| region_td | Regional war exposure cluster | 233.65 | 1 | <0.001 | Time-dependent |
| rcpt_td | Take-home dosing status | 21.66 | 1 | <0.001 | Time-dependent |
| dose_td | Optimal OAMT dose status | 2.53 | 1 | 0.112 | Time-dependent |
| region_perf | Regional OAMT program performance classification | 16.11 | 1 | <0.001 | Time-dependent |
| pop_q | Regional population quartile | 113.11 | 1 | <0.001 | Time-dependent |
| grdp_q | Regional gross regional domestic product quartile | 80.54 | 1 | <0.001 | Time-dependent |
| age1 | Age category | 5.19 | 1 | 0.023 | Baseline |
| sex1 | Sex | 3.08 | 1 | 0.079 | Baseline |
| drug | Medication type | 7.06 | 1 | 0.008 | Time-dependent |
| ep | Treatment episode number | 1.00 | 1 | 0.318 | Baseline |
| dur | Treatment duration | 11.44 | 1 | <0.001 | Baseline |
| idu1 | Injection drug use history | 3.04 | 1 | 0.081 | Baseline |
| hiv_stat | HIV status | 0.08 | 1 | 0.778 | Baseline |
| GLOBAL | Overall model test | 581.89 | 13 | <0.001 | Baseline |

The proportional hazards assumption was evaluated using Schoenfeld residual tests and visual inspection of scaled Schoenfeld residual plots for all covariates included in the final Cox regression model (Figure A3). As shown in Table A3, the global Schoenfeld residual test was statistically significant (χ²=581.89, df=13, p<0.001), indicating evidence of overall non-proportionality across the model. The strongest statistical deviations from proportional hazards were observed among the time-dependent war exposure and regional contextual variables, particularly regional war exposure (region_td), population quartile (pop_q), and regional GDP quartile (grdp_q), all of which demonstrated highly significant time-varying effects over follow-up. Additional evidence of moderate non-proportionality was observed for take-home dosing (rcpt_td) and regional OAMT program performance (region_perf). Among baseline variables, treatment duration (dur) and medication type (drug) demonstrated moderate evidence of non-proportional hazards, whereas dose category (dose_td), treatment episode number (ep), sex (sex1), injection drug use history (idu1), and HIV status (hiv_stat) demonstrated no substantial evidence of proportional hazards violations.

Visual inspection of the scaled Schoenfeld residual plots (Figure A3) demonstrated that most deviations were gradual and monotonic rather than severe or highly nonlinear. The clearest time-varying trends were observed for regional war exposure and regional socioeconomic indicators, which demonstrated gradual shifts in effect estimates over follow-up. Importantly, no covariate demonstrated dramatic instability, sharp nonlinear effects, or major departures from expected model behavior. Because the analysis incorporated time-dependent covariates within a counting-process survival framework and modeled evolving wartime conditions over time, moderate departures from strict proportional hazards assumptions were anticipated and considered methodologically acceptable.

**Figure A3. Scaled Schoenfeld Residual Plots for Assessment of the Proportional Hazards Assumption in the Final Cox Regression Model**

V. **Pre-War Regional OAMT Performance Categorization Variable Construction**

Pre-war regional OAMT program performance categories were initially derived using two-step cluster analysis based on regional NIATx implementation characteristics, including collaboration climate, leadership participation, risk-taking orientation, participation in NIATx activities, and implementation-related engagement measures collected for the separate study **(See Table A4).**^1^ Subsequent longitudinal analyses (2017-2021) demonstrated that the identified regional clusters differed significantly in both patient acquisition and dropout rates over time. In generalized linear models, acquisition rates varied significantly across clusters (**See Figure A4**), with the “High acquisition/low DO dropout” group demonstrating the highest acquisition levels, followed by the “Mid-level acquisition/high DO dropout” and “Low acquisition/mid-level DO dropout” groups. The multivariate general linear model (GLM) procedure, with Acquisition Rate as the dependent variable, and Cluster and Month as independent variables. Results revealed main effects for Cluster (F =67.1, η2 =.71) and for Month (F =4.49, η2 =.67), p <.01 in both cases. Turkey’s HSD pair-wise comparisons among the three clusters were significant (p < .01 in all cases). Similarly, dropout rates differed significantly between clusters (F=59.53, η²=0.62, p<0.001), with the “Mid-level acquisition/high DO dropout” group exhibiting the highest dropout burden (**See Figure A5**). The GLM procedure with the Drop Rate as the dependent variable, and Cluster and Month as independent variables, produced one main effect for Cluster (F=59.53, η2 =.62, p < .01), but not for Month (p = .528). The differences among clusters were significant (p < .01 in all cases).

This variable was constructed to account for baseline regional differences in pre-war OAMT program performance and implementation capacity and was included in the war-related survival models as a region-level adjustment factor.

**Table A4. Ukraine’s Regions and Corresponding NIATx Measures**

| **Region** | **Trip** | **Collaboration Climate** | | **Risk-taking** | **Staff Participation** | **CN Participation** | **Cluster** |
| --- | --- | --- | --- | --- | --- | --- | --- |
|  | 0=no trip, 1=Trip1,  2=Trip2 | Scale Sum | Quartile: 1=below 25% to 4=above 75% | 1=low, 2=mixed, 3=high | 1=high,  2=low | 1=high,  2=low | 1=Engaged Collaborators, 2=Risk-takers, 3=Laggers |
| Vinnytsya | 2 | 11.14 | 2 | 2 | 1 | 1 | 2 |
| Volyn | 0 | 12.29 | 3 | 1 | 0 | 0 | 3 |
| Dnipropetrovsk | 1 | 10.39 | 1 | 2 | 1 | 1 | 2 |
| Donetsk | 2 | 11.79 | 2 | 2 | 0 | 0 | 3 |
| Zhytomyr | 2 | 6.86 | 1 | 2 | 0 | 0 | 2 |
| Zakarpattya | 0 | 14.25 | 4 | 1 | 0 | 0 | 3 |
| Zaporizhya | 2 | 10.96 | 1 | 1 | 1 | 0 | 2 |
| Ivano-Frankivsk | 1 | 13.75 | 4 | 2 | 1 | 1 | 1 |
| Kirovohrad | 2 | 11.00 | 2 | 1 | 1 | 1 | 2 |
| Kyiv city | 1 | 11.93 | 3 | 3 | 1 | 1 | 1 |
| Kyiv region | 0 | 13.50 | 4 | 1 | 0 | 0 | 3 |
| Lugansk | 0 | 9.96 | 1 | 1 | 0 | 0 | 3 |
| Lviv | 2 | 12.00 | 3 | 3 | 0 | 1 | 1 |
| Mykolaiv | 1 | 11.00 | 2 | 2 | 1 | 1 | 2 |
| Odessa | 0 | 8.82 | 1 | 1 | 1 | 1 | 3 |
| Poltava | 2 | 13.75 | 4 | 3 | 1 | 1 | 1 |
| Rivne | 0 | 11.46 | 2 | 1 | 0 | 0 | 3 |
| Sumy | 1 | 14.43 | 4 | 1 | 1 | 1 | 1 |
| Ternopil | 0 | 12.32 | 3 | 1 | 0 | 0 | 3 |
| Kharkiv | 0 | 12.64 | 3 | 1 | 1 | 1 | 1 |
| Kherson | 2 | 12.86 | 3 | 1 | 1 | 1 | 1 |
| Khmelnytsk | 0 | 11.71 | 2 | 1 | 0 | 0 | 3 |
| Cherkasy | 2 | 11.25 | 2 | 2 | 0 | 0 | 2 |
| Chernivtsi | 0 | 3.00 | 1 | 1 | 0 | 0 | 3 |
| Chernihiv | 2 | 13.25 | 4 | 1 | 1 | 1 | 2 |

**Figure A4.** Acquisition Rate by Month by Cluster (2017-2021).

**Figure A5.** Drop Rate by Month by Cluster (2017-2021).

References

1.	Madden L, Ivasiy R, Polonsky M, et al. Scaling up Opioid Agonist Therapies in Ukraine Before the War: Implementation Factors Related to Disruptions From Legislative Reform and COVID-19. *Drug and Alcohol Dependence*. 2024;260:110714.

**IV. Sensitivity Analysis**

This model uses the revised dropout definition: dropout was defined as an interruption in OAMT lasting 30 or more consecutive days, rather than the prior 10 or more day interruption threshold.

Supplementary Table S2. Sensitivity Analysis Using a 30-Day Definition of OAMT Dropout

| **Characteristic** | **Category** | **aHR** | **Lower 95% CI** | **Upper 95% CI** | **p-value** |
| --- | --- | --- | --- | --- | --- |
|  | Low-Intensity Local | Ref. |  |  |  |
| War Intensity Local Displacement Status | High Intensity Local | 5.28 | 4.06 | 6.88 | <0.001 |
|  | Frontline Local | 11.30 | 9.05 | 14.12 | <0.001 |
|  | Low-Intensity IDP | 3.95 | 3.22 | 4.85 | <0.001 |
|  | High-Intensity IDP | 16.56 | 12.20 | 22.47 | <0.001 |
|  | Frontline IDP | 19.99 | 12.07 | 33.09 | <0.001 |
| Medication Type | Methadone (MET) | Ref. |  |  |  |
|  | Buprenorphine (BUP) | 1.14 | 1.04 | 1.25 | <0.001 |
| Take-home Dosing | Daily supervised dosing | Ref. |  |  |  |
|  | Unsupervised dosing | 0.70 | 0.63 | 0.78 | <0.001 |
| Optimal Dose Status | Not optimal dose | Ref. |  |  |  |
|  | Optimal dose | 0.80 | 0.75 | 0.85 | <0.001 |
| Pre-war Regional OAMT Performance | High acquisition/low dropout | Ref. |  |  |  |
|  | Mid-level acquisition/high dropout | 1.19 | 0.99 | 1.43 | 0.07 |
|  | Low acquisition/mid-level dropout | 1.26 | 1.07 | 1.48 | 0.01 |
| Population Quartile | Q1 (<0.9 million) | Ref. |  |  |  |
|  | Q2 (0.9–1.2 million) | 1.21 | 0.99 | 1.47 | 0.06 |
|  | Q3 (1.2–1.4 million) | 0.37 | 0.28 | 0.49 | <0.001 |
|  | Q4 (>1.4 million) | 0.49 | 0.37 | 0.64 | <0.001 |
| Gross Regional Domestic Product Quartile | Q1 (<0.5K–1.7K) | Ref. |  |  |  |
|  | Q2 (1.7K–2.1K) | 1.12 | 0.89 | 1.42 | 0.33 |
|  | Q3 (2.2K–2.8K) | 0.91 | 0.72 | 1.15 | 0.43 |
|  | Q4 (>2.8K–4K) | 0.48 | 0.34 | 0.67 | <0.001 |
| Age, years | <34 years | Ref. |  |  |  |
|  | ≥34 years | 0.93 | 0.86 | 1.00 | 0.05 |
| Sex | Male | Ref. |  |  |  |
|  | Female | 1.17 | 1.07 | 1.27 | <0.001 |
| Treatment Episode | First OAMT episode | Ref. |  |  |  |
|  | Repeat OAMT episode | 1.41 | 1.30 | 1.52 | <0.001 |
| Treatment Duration | <1 year | Ref. |  |  |  |
|  | 1–3 years | 0.87 | 0.73 | 1.05 | 0.14 |
|  | >3 years | 0.66 | 0.56 | 0.78 | <0.001 |
| Injection Drug Use | No | Ref. |  |  |  |
|  | Yes | 0.96 | 0.89 | 1.04 | 0.28 |
| HIV Status | HIV-negative | Ref. |  |  |  |
|  | HIV-positive | 1.04 | 0.97 | 1.11 | 0.31 |
